# Organizational Neuroscience of Industrial Adaptive Behavior

**DOI:** 10.3390/bs12050131

**Published:** 2022-05-03

**Authors:** Stephen Fox, Adrian Kotelba

**Affiliations:** VTT Technical Research Centre of Finland, FI-02150 Espoo, Finland; adrian.kotelba@vtt.fi

**Keywords:** adaptive behavior, competition, ecological fitness, entropy, environment, inference, lock-ins, organizational behavior, organizational neuroscience, signaling

## Abstract

Organizational neuroscience is recognized in organizational behavior literature as offering an interpretive framework that can shed new light on existing organizational challenges. In this paper, findings from neuroscience studies concerned with adaptive behavior for ecological fitness are applied to explore industrial adaptive behavior. This is important because many companies are not able to manage dynamics between adaptability and stability. The reported analysis relates business-to-business signaling in competitive environments to three levels of inference. In accordance with neuroscience studies concerned with adaptive behavior, trade-offs between complexity and accuracy in business-to-business signaling and inference are explained. In addition, signaling and inference are related to risks and ambiguities in competitive industrial markets. Overall, the paper provides a comprehensive analysis of industrial adaptive behavior in terms of relevant neuroscience constructs. In doing so, the paper makes a contribution to the field of organizational neuroscience, and to research concerned with industrial adaptive behavior. The reported analysis is relevant to organizational adaptive behavior that involves combining human intelligence and artificial intelligence.

## 1. Introduction

In neuroscience research, it has been argued that access to a higher number of neural states can better facilitate adaptation with changing environments. It has been argued that access to a higher number of neural states can be described as higher brain entropy [1]. This is a latent entropy, as it refers to the number of neural states that could be accessed. Apropos, brain entropy is associated with potential for divergent thinking [2]. Yet, it is also recognized that neurological functioning needs to be efficient [3], and that actual entropy needs to be minimized [4].

For example, if an industrial organization offers too many product variations, its production operatives may experience information-theoretic entropy from information uncertainty about how to carry out production processes. This information uncertainty can lead to the statistical mechanics entropy of physical disorder in production as operatives attempt several different ways to carry out the work. For example, if there is an information-theoretical entropy of 2.58 bits, there are six equiprobable but different ways in which a task could be carried out. If only one of those six different ways of carrying out the work is correct, there will be thermodynamic entropy when the production operatives’ potentially useful thermodynamic energy becomes practically useless thermodynamic energy as it is lost in failed actions to carry out work [5]. Accordingly, the balancing of adaptability and stability depends upon balancing latent organizational entropy for adaptability, and actual process entropy for stability. This balancing of adaptability and stability is necessary for an organization to be viable [6]. In this paper, comprehensive analysis is provided of industrial adaptive behavior in competitive markets. The analysis draws upon studies founded upon theoretical neuroscience concerned with adaptive behavior for ecological fitness. Within these studies, living things have internal generative models that need to be synchronized with external generative processes in the environment. External generative processes and internal generative models should have reciprocal exchanges of signals and inferences that inform reciprocal learning and development. Synchronization needs to balance trade-offs between complexity and accuracy to achieve futures that are minimally risky and minimally ambiguous [4,7,8,9,10,11].

Organizational neuroscience is recognized in the organizational behavior literature as offering an interpretive framework that sheds new light on existing problems [12]. The analysis presented in this paper is novel in that it provides three interrelated contributions to organizational behavior research. First, it extends the application of organizational neuroscience in the analysis of interactions between organizations. By contrast, other organizational neuroscience studies have focused on behavior within organizations, for example leadership within organizations [13], and management decision-making within organizations [14]. The second contribution is to relate organizational neuroscience to management literature concerned with organizational adaptive behavior [6,15]. The third contribution is to relate organizational neuroscience concerned with adaptive behavior to industrial practice. This is important as most industrial organizations continue to be either agile through focusing on adaptability or lean through focusing on stability [16].

The analysis of industrial adaptive behavior is presented in six further sections. In Section 2, neuroscience studies are related to efforts in industry to balance adaptability and stability. In Section 3, analysis of signaling and inference between industrial companies is provided. In Section 4, trade-offs between complexity and accuracy in industrial adaptive behavior are explained. In Section 5, industrial adaptive behavior is related to risks and ambiguities in competitive environments. In Section 6, principal contributions are stated, practical implications are discussed, and directions for future research are proposed. In Section 7, conclusions are stated.

## 2. Adaptability/Stability Dynamics

In practice, industrial software systems, such as product configurators, can contribute to mediating between adaptability and stability. Product configurators can be described as online brochures, which enable potential end-users to select and configure sub-assemblies into their preferred products [17]. In terms of signaling, product configurators signal fittest offers by displaying product options and receive signals of end-user preferences by receiving orders for product options. To continue with this framing, product configurators generate patterns of interaction with the world based on an organization’s generative model. For practical purposes, the generative models of industrial organizations are their business models [18]. In the short-term, internal stability can be facilitated by not updating the variety of signaling with product configurators. However, not updating signaling variety can lead to insufficient adaptation to environmental changes in competitive markets. This can be framed as under-fitting an organization’s generative model to the competitive environment. Conversely, continually updating signaling variety in response to every market signal can lead to over-fitting an organization’s generative model to the competitive environment [19]. This can undermine internal stability as different parts have to be bought in and work processes have to be modified for every contract. Thus, industrial organizations seek to find balance between being as open as possible to market signals while maintaining internal stability by not being too open. This involves common initiatives, such as organizations attempting to transition from engineering-to-order whatever each individual customer may have in mind to mass customization of predetermined sub-assemblies and configuration options. Yet, despite such initiatives to balance adaptability and stability being common, they are seldom entirely successful [20].

In neuroscience research, it has been argued that access to a higher number of neural states can better facilitate adaptive behavior [1,2]. In industrial practice, a company’s number of accessible states can be related to so-called value constellations [21]. For example, value constellations can comprise an original equipment manufacturer’s long list of potential suppliers. Original equipment manufacturer (OEM) is a term used to describe organizations that develop and market manufactured goods [22]. The larger the number of suppliers on the list, the larger the number of supply states that an OEM could access. Nonetheless, when organizational latent entropy is a list of suppliers, organizational latent entropy can be maximized by the company with minimal actual entropy and so with least action [5]. For example, suppliers could submit their details to the OEM via the OEM’s website. Such procedures are consistent with the principle of least effort in the management of information [23,24]. Thus, the value constellation does not entail actual information uncertainty that leads to physical disorder that involves useless expenditure of thermodynamic energy in unproductive actions. Rather, the value constellation has latent entropy, i.e., entropy that is not manifesting but has the potential to do so. From a long list of suppliers, individual project-specific arrangements of suppliers can be made. Project-specific arrangements involve carrying out work to change inter-organizational connections from weak network ties to strong project ties [21]. Such work can involve carrying out negotiations and signing a project contract. If project work proceeds to continuous work, such as when a relationship in product development becomes a relationship in product supply, further work will be carried out to minimize entropy. This can begin with the rationalization of supply chains. Thus, there can be an expansion of the number of latent states through the exploration of potential suppliers, followed by reduction to a few actual supply states that exploit strong ties between OEM and selected suppliers. Yet, the entrenching of supply chain relationships can lead to increasing stability at the expense of adaptability to environmental change [25].

More generally, increasing stability at the expensive of adaptability can involve organizational lock-ins to existing paths of action. The development of organizational lock-ins can involve some paths of actions becoming deeply entrenched until all of the organization’s actions are path-dependent. This can lead to organizations experiencing so-called counterfactual stability due to the organization having what has been described as a rationality shift [26,27,28]. This can involve organizations stubbornly continuing to use old beliefs to try to navigate in new environments [11,29,30]. Organizational commitment to out-of-date paths of action can increase even through there is increasing evidence that doing so is not successful [31,32,33]. Especially when considering itself under threat, an organization can become rigid in its persistence with ineffective actions [34]. Although counterproductive, such commitment to failing courses of action can be seen as a way to manage trade-offs between complexity and accuracy in order to minimize risk and ambiguity. This can happen if organizations seek to reduce complexity by paying more attention to out-of-date beliefs than to signals that indicate the course of action is counterproductive. As a consequence, an organization can cease to be viable because it focuses on internal stability at the expense of its adaptability to environmental change [5].

In terms of neuroscience concerned with adaptive behavior [4,7,8,9,10,11], insufficient adaptation involves an organization’s internal generative model not maintaining synchrony with generative processes in the external states of the world. This could begin with one generative model parameter not maintaining synchrony, which can lead to an organization’s overall generative model losing synchrony. For example, one business model parameter can be product development, and organizations can continue with new versions of old products. This can then lead to loss of synchrony on the product development parameter, which leads to loss of synchrony on the marketing parameter, and so on. Overall, lock-ins can prevent organizations’ generative models from going through necessary cycles of expansion for strategic adaptation, and reduction for operating efficiency [35]. In particular, this may involve expansion to encompass new hypotheses for explaining new signals from the environment, and subsequent reduction by merging several hypotheses into one generalizable new explanation for many new signals. Organizational lock-ins, for example, in terms of products and/or suppliers, can be conceptualized as open slots in a generative model being replaced permanently by fixed chunks. For example, a frame and slot for product development in a generative model can be as follows: a good production vehicle for the market today is X, where X is the open slot. By contrast, a fixed chunk for product development in a generative model can be as follows: a good production vehicle for the market today is always our old production vehicle that was the market leader for many years. If this happens, organizations can confuse what they imagine based on memories with the operating realities of current situations [36]. In such cases, generative models can be scored internally based on the past rather than on synchrony with present environmental conditions. This could contribute to organizations persisting with the signaling of out-of-date products, for example with product configurators and traditional brochures, and suffering reduced customer loyalty due to a lack of innovation [37,38,39,40].

## 3. Signaling and Inference

**Figure 1:**
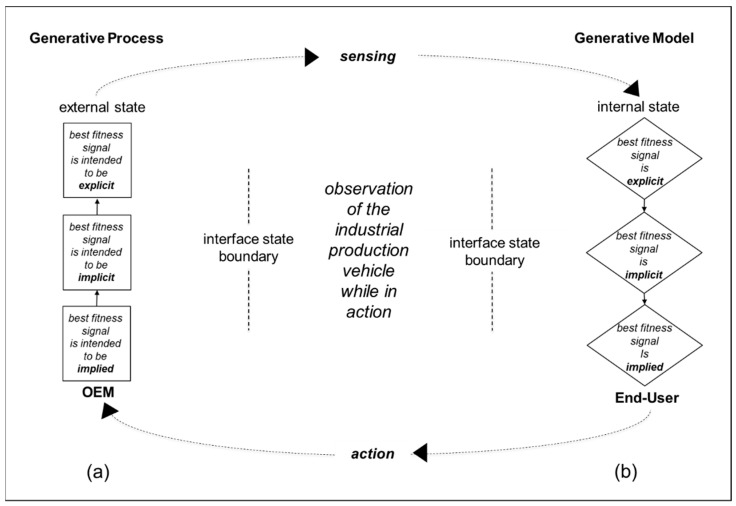
Inter-organizational signaling. (**a**) The developer/vendor (OEM) of a production vehicle intends that its signal of best ecological fitness will be implied, implicit, and explicit. (**b**) User organization observes that the production vehicle does provide explicit, implicit, and implied best fitness.

Figure 1 shows exchanges between generative processes in the external states of the world and generative models in internal states of an organization. In this example, an OEM is the source of the generative processes in the external state of the world, and the generative model is that of an end-user company. Figure 1 is an original diagram. However, distinguishing between external states and internal states is fundamental in neuroscience research concerned with adaptive behavior for ecological fitness [7,8,9,10,11], and is consistent with well-established conceptualizations in organizational studies. For example, interactions between internal states and external states are important in Kurt Lewin’s field theory [41]. Additionally, interactions between internal states and external states are important in Ronald Coase’s article “The Nature of the Firm”. In particular, where companies position their boundaries can depend upon a comparison of differences between the transaction costs of doing work internally or buying in work done in the market [15]. Here, end-users are the users of the manufactured goods.

Generative processes in external states can cause the sensory inputs of agents. For example, the sensory inputs of agents can be caused by product signals [42,43]. In Figure 1, the agent is the end-user. Agents’ generative models provide the basis for inferences and for generating patterns of interaction with external states. Sensing and actions take place in the interface state. The greater the synchronicity in exchanges between generative processes and generative models, the greater the ecological fitness of the agent and the potential for long-term survival. Here, synchronicity refers to back-and-forth reciprocal exchanges of learning and development [11,44]. In the example summarized in Figure 1, the OEM develops and markets large industrial production vehicles, while the end-user leases and operates one of the OEM’s large industrial production vehicles. As summarized in Figure 1, the OEM begins its product development process from what is intended to be implied and works towards what is intended to be explicit. In particular, the starting point for the product design is the developer’s brand. From this basis, product design proceeds from concept design through to detail design of product shape and features [45,46,47]. This is done with the intention of the shapes of product features signaling meanings to end-users that differentiate them positively from competitors’ offerings [48,49].

**Figure 2:**
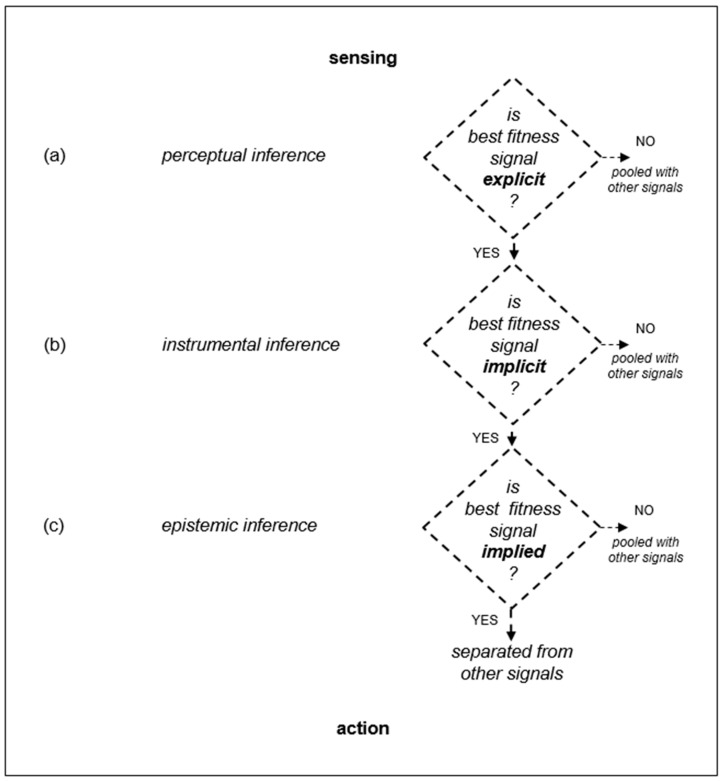
Multi-level inference of signals. (**a**) Perceptual inference of whether or not there is an explicit offer of best ecological fitness: if yes, inference proceeds; if no, the signal is pooled with other signals. (**b**) Instrumental inference of whether or not there is an implicit offer of best ecological fitness: if yes, inference proceeds; if no, signal is pooled with other signals. (**c**) Epistemic inference of whether or not there is an implied offer of best ecological fitness: if yes, signal is separated positively from other signals; if no, signal is pooled with other signals.

The use of the production vehicle takes place in the interface state between what is the external state from the point of view of the end-user, and the end-user organization’s own internal state. Here, for practical purposes, the interface state can be thought of as a work site situated somewhere between the OEM’s premises and the end-user’s premises. Figure 2 illustrates a situation where the OEM’s intended signaling of implied, implicit, and explicit fitness offer to the end-user is matched by the actual fitness provided to the end-user from its operation of the production vehicle. In particular, the production vehicle offers the best fitness by enabling the organization to be more successful than its rivals in the competitive environment in which it intends to survive.

Figure 2 is an original diagram that shows a hierarchical arrangement of perceptual inference, instrumental inference, and epistemic inference. This is consistent with hierarchical schemes of inference in neuroscience [13], and with the ladder of inference in organizational studies [50]. In Figure 2a, perceptual inference refers to the ability to infer sensory stimuli from predictions that result from internal representations built through prior experience [51,52], which can begin with observation of a product’s physical features as they come into view, or as it is brought into view. For example, this might include perceptual inference of an industrial production vehicle’s physical features. In Figure 2b, instrumental inference refers to inference about actions in a competitive environment. For example, this might mean that an industrial production vehicle’s physical features are indicative of its capabilities to carry out actions needed to survive in the competitive environment. In Figure 2c, epistemic inference refers to inference concerned with beliefs about how to survive in a competitive environment. For example, an industrial production vehicle’s features can imply that it is probably the most versatile production vehicle, and can best enable survival amidst competition [53,54].

In addition, the diagram in Figure 2 illustrates that there can be observations of many product signals, and product signals that are not separated from other product signals by explicit, implicit, and implied offers of the best fitness will be pooled together rather than providing a basis for action [55]. Consider, for example, that the end-user shown in Figure 1 receives many brochures for production vehicles from different OEMs. Following perceptual, instrumental, and epistemic inference, the end-user takes the action of leasing the production vehicle shown in Figure 1 OEM’s brochure. The end user’s generative model, e.g., its business model [18], now incorporates the end-user taking action with the production vehicle. The other brochures from the other OEMs are put together for future reference if necessary (i.e., the other brochures and the signals that they entail are in a pooling equilibrium). In other words, observers pay more attention to differentiated signals than to those that are not easily differentiated from each other. As summarized in Figure 1, the end-user’s ecological fitness in its competitive environment is facilitated by leasing and operating the selected OEM’s production vehicle. Consequently, it is not pooled with the production vehicles described in other OEM’s brochures. Rather, it stays positively separated from them. Hence, the production vehicle continues to be leased and operated.

## 4. Complexity versus Accuracy

**Figure 3:**
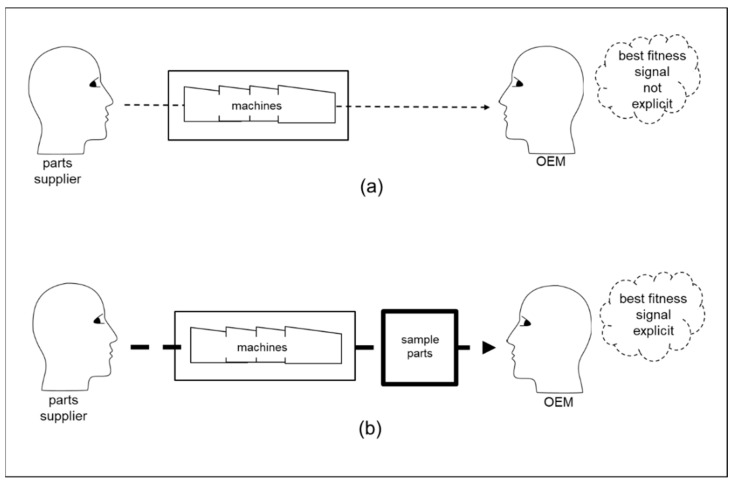
Complexity/accuracy trade-off. (**a**) The OEM’s model is too complex because the OEM has multiple inferential steps in trying to relate manufacturing facilities to the parts to be manufactured. Hence, the OEM’s prediction accuracy about the parts supplier is not well enabled. (**b**) The OEM’s model is less complex because the OEM has fewer inferential steps due to the parts supplier having made explicit what was implicit and implied in (**a**) by investing in sector-specific component samples as well as premises and machines. Hence, the OEM’s prediction accuracy is facilitated.

In the example summarized in Figure 1, the OEM is in the external state from the point of view of the end-user. However, from the point of view of a parts supplier to the OEM, the OEM is in the internal state. Interactions between the three organizations can be summarized linearly as follows: parts supplier–OEM–end-user. In this next example, the focus is as follows: parts supplier–OEM. The OEM has a generative model that encompasses parts suppliers, which provide its basis for interpreting signals from parts suppliers and for generating patterns of interaction with parts suppliers. The OEM wants its predictions about what will happen during interactions with parts suppliers to be as accurate as possible. For example, it wants it predictions of supplier performance to be accurate. Yet, at the same time, the OEM does not want its generative model to be very complex. Here, complexity can be considered in terms of the number of inferential steps that the OEM needs to take to update its generative model based on signals from parts suppliers. Generative synchronicity depends upon back-and-forth reciprocal exchanges of learning and development. However, this may not be straightforward for parts suppliers. This is because part suppliers often do not develop the machines that they use to manufacture the parts that they supply. Additionally, they often do not design the parts that they manufacture. One option is for parts suppliers to try to signal the best fitness offer to the OEM by increasing the size of their premises and buying in more machines in order to set up sector-specific production areas. These are investments that can enable the parts supplier to offer better parts more quickly to OEMs in different sectors, which in turn can enable the OEMs to be more competitive. As summarized in the original diagram in Figure 3, there can be two scenarios involving different levels of financial investment for the parts supplier. In scenario (a) the company uses its newly developed capabilities from its investments in premises and machines as the basis of its signaling to OEMs. However, scenario (a) leaves the parts supplier in an unfavorable pooling equilibrium with other parts suppliers. Therefore, in scenario (b) the company uses its newly developed production capabilities to manufacture at its own cost samples that are typical of the types of parts required by the OEM. These samples are exemplary of part features that are both performance-critical and difficult to manufacture. In scenario (b), the parts supplier uses exemplary parts as the basis of its signaling. This practical investment in exemplary physical samples extends the bases for signaling [55] and provides maximum relevant information [56]. Importantly, scenario (b) reduces the number of inferential steps required by the OEM to update its generative model of parts suppliers. Hence, the OEM can more easily update its generative model to include the parts supplier. This is because the parts supplier demonstrates production samples that are both performance-critical and difficult to manufacture, rather than just premises and machines as in scenario (a).

Scenario (b) involves the parts supplier making explicit in exemplary samples what was implicit and implied, by increasing the size of its premises and in buying more machines. Thus, in scenario (b), the parts supplier’s behavior is consistent with the optimal signaling strategy of presenting its most favorable trait for sensing by the OEM in its first signaling [56,57]. This example illustrates that adaptations in generative processes can decrease the complexity of generative models and increase the accuracy of predictions from generative models. In nature, such adaptations can take millennia. By contrast, as illustrated by the parts supplier example, human organizations in the generative process of the external state can adapt rapidly, for example in a few months, to develop clear positive signaling of best fitness that decreases the complexity and increases the accuracy of generative models in the internal state. This is consistent with the cognitive niche theory that combinations of human cognitive adaptive behavioral traits have evolved in order to facilitate competitive adaptation on faster time scales than in natural evolution [58,59].

From the point of view of the OEM, the parts supplier’s action in scenario (b) conforms to the principle of least effort [23,24]. In particular, it conforms to the preference for least effort in instrumental inference for particular supplier contracts, and least effort in epistemic inference to update its beliefs about suppliers. However, in accordance with the principle of least collaborative effort in pragmatics [60,61], signalers with high fitness may seek to incur the least cost in separating themselves from signalers with lower fitness. Yet, high-fitness signalers want to avoid being in a pooling equilibrium with low-fitness signalers. Moreover, signalers with lower fitness may choose to incur higher signaling costs in order to put themselves in a pooling equilibrium with high-fitness signalers [62,63,64]. Thus, there can be dynamic motivations for organizations to sometimes make explicit what could be implicit and implied, but at other times to seek to gain competitive advantage from leaving much implicit and implied. For example, some organizations can choose to be very understated in signaling unobservable product qualities when being very subtle in communicating their brand identity [65,66]. Thus, reducing complexity and increasing accuracy in generative synchronicity can be a dynamic challenge as adaptations in the competitive environment adapt organizations, and then organizations adapt the competitive environment in back-and forth-reciprocal exchanges of learning and development [11,44].

## 5. Risk and Ambiguity

**Figure 4:**
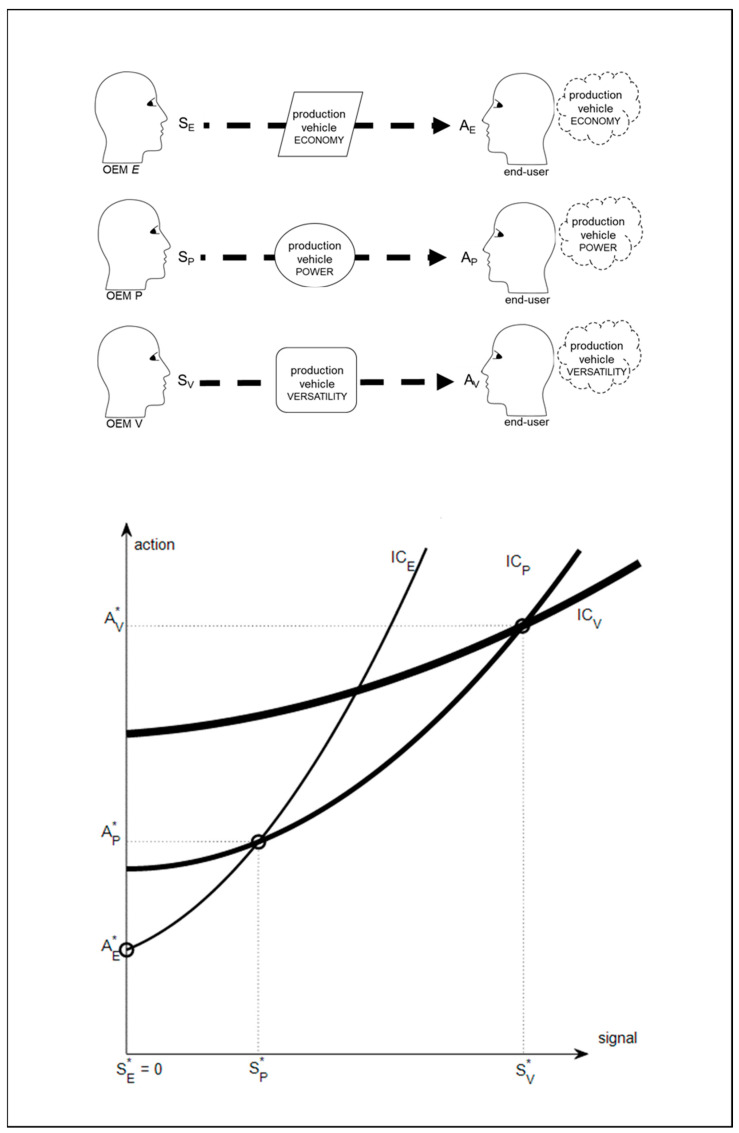
Generative synchronicity in an oligopoly. Three OEMs, E, P, and V, signal different fitness offers to end-users through primarily emphasizing the economy (E) or the power (P) or the versatility (V) of their production vehicles in accordance with the preferences of end-users.

Generative synchronicity can entail coupling between generative processes in external states and generative models in internals states. However, environmental perturbations can disturb coupling and undermine generative synchronicity. In this case, agents need to focus on minimizing risks to their survival from their interactions with the external state and minimize the ambiguity of observations that could lead them to overestimate or underestimate risks. Consider that, as illustrated in the original diagram in Figure 4 below, the OEM in the examples above is in a global oligopoly of large production vehicle OEMs. Each of the three OEMs in the oligopoly, E, P, and V, signals different fitness to end-users through primarily emphasizing the economy (_E_), or the power (_P_), or the versatility (_V_) of their production vehicles in accordance with the preferences of end-users. The OEM in the examples above and in the following example is OEM V. An oligopoly can be described as a market in which sellers are so few that the actions of any one of them will affect price and impact on competitors. Figure 4 includes indifference curves (IC), as in [67]. These indicate the scope of potential interactions between signals and actions in the opinion of the respective OEMs E, P, and V. In other words, IC_E_ for OEM E, IC_P_ for OEM P, and IC_V_ for OEM V. To begin with, all potential interactions that lie on the indifference curve are considered to be equally useful by OEMs. This is because it is possible that any point on the indifference curve could provide separating equilibrium. However, signal S*_E_ attracts action A*_E_ from end-users with preference for economy, signal S*_P_ attracts action A*_P_ from end-users with preference for power, and signal S*_V_ attracts action A*_V_ from end-users with preference for versatility. Hence, a separating equilibrium emerges.

**Figure 5:**
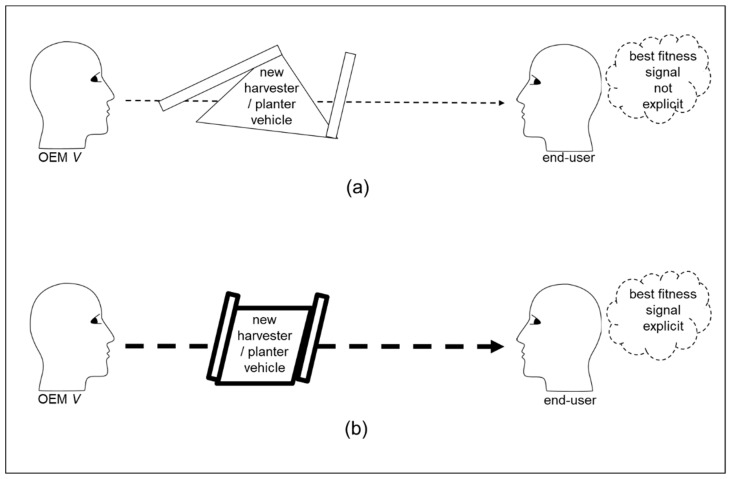
Risk and ambiguity. (**a**) The end-user assesses the risk to be higher because the end-user has many inferential steps due to the OEM developing a new harvester/planter vehicle that is not symmetrical and does not have the easily recognizable features of the OEM’s previous product vehicles. (**b**) The end-user assesses the risk to be low because the end-user has fewer inferential steps due to the OEM developing a new harvester-planter vehicle that is symmetrical and does have easily recognizable features of the OEM’s previous product vehicles.

However, the oligopoly can be disturbed when global recession leads to a reduced global demand for production vehicles. This leads to the OEMs in the oligopoly having to try to increase their shares of a shrinking global market. One or all of the three OEMs may seek to increase market share through adaptation involving developing a vehicle that is innovative in that it enables planting as well as harvesting. Thus, the production vehicle is visibly different from all other previous and existing production vehicles in that sector of production. As summarized in the original diagram in Figure 5 below, this can lead to two initial scenarios, which can be summarized as (a) unsuccessful scenario, and (b) successful scenario. In the unsuccessful scenario (a), the new harvester-planter vehicle is not symmetrical and it does not have visual features of OEM V‘s previous production vehicles. Hence, the signal from the generative process of external state is ambiguous when observed by end-users. Conversely, in the successful scenario (b), the new harvester-planter vehicle is symmetrical and has many physical features of the company’s previous production vehicles. Hence, the signal from the generative process of external state is not ambiguous when observed by end-users. The unambiguous signal involves end-users undertaking fewer inferential steps because it is congruent with the human preference for symmetrical signals [68] and for the familiar [69,70]. Both of which are congruent with the human preference for making least effort to obtain information [23,24].

In the unsuccessful scenario (a), end-users infer that the company’s offer of best fitness for environments requiring versatility is reduced. This is because the asymmetrical new vehicle has the unintended negative consequence of making OEM V’s established products seem worse [71]. In other words, through the end-users’ instrumental inference, the vehicle’s asymmetry entails the implicit signal that the machine’s physical features are not appropriate for its intended use. Furthermore, through the end-users’ epistemic inference, the vehicle’s asymmetry entails the implied signal that it is not the most versatile production machine of its type, which can best enable fitness. Thus, end-users infer that there would be greatly increased risk from continuing to operate OEM V’s production vehicles.

By contrast, in the successful scenario (b), OEM V changes what end-users believe is required for fitness in the business environment from a harvester vehicle to a combined harvester-planter vehicle. Thus, through their epistemic inference, end-users change their belief from the production vehicles of OEM E and OEM P offering the best fitness to them no longer offering the best fitness. In other words, the explicit physical features of competitors’ vehicles now entail the implicit observation that they are still appropriate for their intended use, but the implied observation that they can no longer enable the best fitness because there is now a combined harvester-planter available. Thus, end-users infer that there would be increased risk from continuing to operate OEM E’s production vehicles or OEM P’s production vehicles.

Hence, in the successful scenario (b), OEM V could expand its market share of the shrinking market sufficiently to maintain turnover and profits, despite having incurred extra product development costs in order to increase fitness. However, hostile responses can be expected from competitors in an oligopoly if they believe that others are taking a disproportionate market share. Thus, OEM E and OEM P will seek to re-establish their typical share as the previous status quo [72]. Here, the so-called quick reaction hypothesis is relevant [67]. This is the assumption that at least one other signaler will try to exploit the opportunity arising from its competitor’s new offering before all possible sales from such a new offering are made. Thus, in accordance with the extended evolutionary synthesis [73], adaptations in the competitive environment adapt the OEM, and then the OEM adapts the competitive environment in back-and-forth reciprocal exchanges of learning and development [11,44]. Importantly, the actions of one signaler, such as OEM *V*, can change what is explicit, implicit, and implied in its own offering of fitness, and adapt what is implied by the offerings of competing signalers.

## 6. Discussion

### 6.1. Principal Contributions

As summarized in Table 1 below, the paper provides a comprehensive analysis of industrial adaptive behavior in terms of neuroscience studies referred to in the preceding sections. The comprehensive analysis goes beyond previous studies [74] in providing three interrelated contributions to the field of organizational behavior. First, it extends the application of organizational neuroscience in the analysis of interactions between organizations. Second, the analysis brings together organizational neuroscience with relevant management literature concerned with organizational adaptive behavior. The third contribution is to relate organizational neuroscience concerned with adaptive behavior to industrial practice. This is important as many industrial organizations have difficulties in managing adaptability/stability dynamics.

Interplay between adaptability and stability have been related to generative model expansion and reduction. As summarized in Figure 1, signals arise from external generative processes and inferences arise from internal generative models. Explicit signaling is related to perceptual inference, implicit signaling to instrumental inference, and implied signaling to epistemic inference. As illustrated by the examples, the same one organization can provide signals to another organization, such as an end-user, and making inferences about another organization, such as a supplier. The need for signaling to decrease the complexity and increase the accuracy of organizations’ inference has been related to the need for organizations to reduce risks and ambiguities in competitive markets.

Overall, the comprehensive analysis summarized in Table 1 has scientific implications for research concerned with inter-organizational behavior that involves both human intelligence and artificial intelligence. For example, generative models, generative processes, and the interplay between them are directly relevant to increasingly commonplace cyber-physical systems that are managed by so-called digital twins. These are digital models of physical processes that are connected to physical processes through sensors and actuators. Digital twins are intended to carry out analyses of physical processes in order to improve their performance [75,76,77]. Importantly, model complexity versus model accuracy, process risk and process ambiguity are applicable to digital twins and to physical processes that exchange signals and inferences to improve performance. Moreover, neuroscience studies of generative model expansion and reduction are relevant to organizations that may need to avoid human lock-ins to out-of-date practices, which could be even more challenging to overcome if they are embedded within digital twins.

### 6.2. Practical Implications

A practical implication is that organizations can benefit from conceptualizing their offerings in terms of functional fitness components and signaling fitness components [78]. For example, as summarized in Figure 3, a parts supplier company investing in improving functional fitness components, such as production premises and production machines, is not sufficient for OEMs to infer easily an offer of best fitness from the parts supplier. Rather, investment in signaling components may be necessary. For example, this might involve investment in the manufacture and presentation of exemplary samples. However, the parts supplier’s signaling components need to be aligned with the OEM’s internal generative model by which OEM generates its inferences and patterns of interaction with the world. Similarly, OEMs need to align their signaling with the generative models of end-user organizations. As summarized in Figure 5, failure to do so can lead to end-user organizations inferring that an OEM’s former offer of best fitness has been lost instead of a new best fitness being offered. Furthermore, organizations need to maintain the potential for the expansion of their generative models. This is necessary to enable long-term survival through adaptive behavior in competitive environments.

### 6.3. Directions for Future Research

One direction for further research is to relate findings from neuroscience research concerned with generative model expansion [35] to organizational research concerned with contingency planning in times of success for potential challenges in the future [79]. This could involve making reference to neuroscience studies concerned with thinking about potential future actions and how they could affect future beliefs [80]. More broadly, further research could address interrelationships between generative model expansion and so-called organizational intelligence [81]. As the loss of signaling capabilities is associated with a loss of potential for adaptive behavior [82], such research could investigate potential contributions to organizational intelligence from improving signaling capabilities from organizations’ generative models [83,84,85]. This research could encompass industrial software systems, such as product configurators. Apropos, one application of neuroscience research would be to investigate to what extent, if any, research into generative model expansion and reduction in synchronization within competitive environments can inform improved implementation of product configurators [86], which can mediate between companies’ adaptability and stability using artificial intelligence [87,88]. This research could include relating previous management studies inspired by neuroscience [6,89] to new findings from neuroscience studies of adaptive behavior as they become available.

## 7. Conclusions

Organizational neuroscience is recognized in the organizational behavior literature as offering an interpretive framework that sheds new light on existing problems. The analysis presented in this paper extends the application of organizational neuroscience in the analysis of interactions between organizations. In addition, organizational neuroscience is related to management literature concerned with organizational adaptive behavior. Furthermore, organizational neuroscience concerned with adaptive behavior is related to industrial practice. Moreover, the main constructs set out in Table 1 with examples are applicable to cyber-physical systems that combine human intelligence and artificial intelligence. This is important because human-artificial intelligence systems are becoming increasingly commonplace and introduce new challenges for organizational behavior.

## Figures and Tables

**Table 1:** Constructs and Examples.

Construct	Description	Examples
Generative process	Causes observations of agents through generation of signals that can be explicit, implicit, and/or implied	OEM generates signals to end-users through explicit product features based on implied brand characteristics
Generative model	Provides basis for interpreting signals and generating patterns of interaction with external states	End-users have generative models for OEMs that encompass the explicit, the implicit, and the implied
Synchronicity	Reciprocal back-and-forth exchanges of learning and development between organization and environment	OEMs’ different offers of fitness to end-users is based on different end-users’ different preferences
Generative model expansion	Generative models can expand to encompass new hypotheses about new causes of new signals	Business models need to expand to enable adaptation to changing markets, but expansion can be restricted by organizational lock-ins
Generative model reduction	Generative models can reduce by merging many hypotheses about causes of many new signals into one hypothesis	Business models need to be rationalized for to enable operating efficiency, while still allowing for future business model expansion
Explicit signals	Sensory stimuli from explicit signals are related by perceptual inference to internal representations built through prior experience	Sensory stimuli, such as light reflecting off vehicle features are related to internal representations of vehicles
Implicit signals	Instrumental inference about what actions to take in the world based can be based on implicit signals	Inference that a production vehicle is appropriate to carry out actions needed to survive in the competitive environment.
Implied signals	Epistemic inference concerned with updating beliefs about the world can be based on implied signals	Inference that a production vehicle is the most versatile production vehicle and can best enable survival amidst competition.
Pooling/Separating	A signal can be pooled with other signals and not acted upon, or a signal can be separated from other signals and acted upon	New signals from OEM V lead to A_E_ and A_P_ to pool signals from OEM E and OEM P
Actions	Actions follow from signals that are positively differentiated from other signals and relate to pre-existing preferences	Different end-users have different preferences for actions with production vehicles: economy, power, versatility
Complexity	The complexity of generative models needs to be minimized to facilitate their efficient reliable updating	Supplier manufactures exemplary parts to make its implicit capabilities explicit and so reduce inferential steps required by OEM
Accuracy	Predictions of interactions with external states from generative model need accuracy to enable synchronicity for long-term survival	OEM cannot make accurate predictions of parts supplier’s performance based on sight of its new premises and production machines
Risk	Agents seek to minimize risk of not being synchronized with external state in order to facilitate long-term survival	During global recession, OEM V seeks to reduce risk for itself and for end-users by introducing planter-harvester vehicle
Ambiguity	Agents seek to minimize the ambiguity of observations that could lead them to underestimate or overestimate risks	Implicit potential of OEM V’s new vehicle to reduce risk is underestimated due to its asymmetrical and unfamiliar explicit design

## Data Availability

Not applicable.
